# Higher Cardio-Ankle Vascular Index Values in Patients With Vestibular Neuritis May Indicate a Better Prognosis

**DOI:** 10.7759/cureus.49151

**Published:** 2023-11-20

**Authors:** Munetaka Ushio, Toshitake Tanaka, Fuyuko Ikemiyagi, Hanako Totsuka, Taro Takanami, Yoshihiro Ikemiyagi, Yoshihisa Kitazawa, Toshiyuki Nomura, Yasushi Ohta, Tomoe Yoshida

**Affiliations:** 1 Otolaryngology, Toho University Sakura Medical Center, Sakura, JPN; 2 Otolaryngology, Ikemi Ear Nose Throat Clinic, Narita, JPN; 3 Otolaryngology, Hiyoshidai Hospital, Tomisato, JPN; 4 Otolaryngology, Kitazawa Ear Nose Throat Clinic, Edogawa, JPN; 5 Otolaryngology, Misato Central General Hospital, Misato, JPN; 6 Clinical Support Service, Toho University Sakura Medical Center, Sakura, JPN

**Keywords:** arteriosclerosis, vascular disorders, etiology, cavi, cardio-ankle vascular index, vestibular neuritis

## Abstract

Background: The presumed etiology of vestibular neuritis (VN), a sudden onset of spontaneous vertigo without auditory or cranial nerve symptoms, includes viral infections and vascular disorders. However, no clinical test for estimating vascular disorders in VN has been reported. Moreover, estimating the etiology of VN is important to predict the prognosis and select appropriate treatment. This study aimed to evaluate the cardio-ankle vascular index (CAVI), which reflects arterial stiffness and elasticity, as an additional indicator for estimating the prognosis and etiology of VN.

Materials and methods: Among 207 consecutive patients with suspected VN, 88 patients diagnosed with definite VN were enrolled. Age, initial and final percent canal paresis (CP) in the caloric test, CAVI, presence or absence of vestibular-evoked myogenic potential asymmetry, and medical history were evaluated using univariate and multivariate analyses.

Results: Patients with VN with high CAVI had a better prognosis than those with low CAVI. High CAVI was a factor for improvement in percent CP, in addition to younger age and less severe initial percent CP in the Cox proportional hazard model.

Conclusion: CAVI can be an additional indicator for estimating the prognosis and etiology of VN. This indicator can potentially be applied to other diseases, including vascular disorders with other etiologies.

## Introduction

Vestibular neuritis (VN) is characterized by a sudden unilateral loss of vestibular function without hearing loss or signs of brain and brainstem lesions [1]. VN has an incidence of 3.5 per 100,000 population [2]. VN etiology in each patient remains unclear, and probable causes include viral infections, such as latent herpes virus simplex type 1 reactivation [3], and vascular disorders, such as vasoconstriction and thrombotic events [4]. The leading hypothesis is latent virus reactivation. However, there are reports with [5] and without [6] enhancement on vestibular nerves suggestive of viral inflammatory findings on gadolinium-enhanced magnetic resonance imaging (MRI). Additionally, antiviral therapy in the acute phase, which was expected, is currently considered ineffective [7]. These results suggest that VN is not a single disease caused by viral infections. To date, no clinical tests have been reported for assessing vascular disorders in this context. More accurate prognosis and etiology assessments should aid in VN prognosis and treatment. Moreover, tests used to identify VN prognosis and etiology may help to identify those of other diseases.

Arteriosclerosis contributes to various vascular disorders, including cardiovascular and cerebrovascular diseases [8]. Arterial stiffness is a property that accompanies the progression of arteriosclerosis [9] and indicates the extent of vascular disorders. Pulse wave velocity (PWV) is a well-known parameter to evaluate arterial stiffness. However, the cardio-ankle vascular index (CAVI) developed in 2006 in Japan is a non-invasive index that reflects arterial stiffness. CAVI is measured by applying cuffs to the bilateral upper arms and ankles with subjects to detect the brachial and ankle pulse waves [10]. CAVI is better for assessing arterial stiffness because, unlike PWV, it is less susceptible to cardiac function and transient blood pressure changes [11]. CAVI is positively correlated with the number of microvascular lesions in the brain [12], such as silent brain infarction, white matter hyperintensities on MRI, and coronary atherosclerosis severity, and the higher the value, the higher the incidence of cardiovascular events [13]. CAVI can thus be employed to evaluate the diseases caused by arterial stiffness.

This is the first report to assess arterial stiffness and elasticity to predict VN prognosis and etiology. We aimed to investigate the correlation between CAVI and the severity and level of improvement in VN to evaluate CAVI as an additional indicator for estimating the VN prognosis and etiology.

## Materials and methods

This retrospective, nonrandomized, single-group study conducted at Toho University Sakura Medical Center was approved by the Institutional Ethics Committee Review Board (S21028). Informed consent was obtained from all patients. The study conformed to the Code of Ethics of the World Medical Association (Declaration of Helsinki).

Participants

Between January 2011 and December 2020, 207 consecutive patients (57.3 ± 17.6 (mean ± standard deviation) years; 92 females and 115 males) were referred to our clinic and suspected to have VN.

VN diagnostic criteria vary among reports. In this study, based on a previous report [5], we set more restrictive criteria for VN diagnosis as follows: (1) history of a single acute or subacute onset of severe, prolonged vertigo attacks for more than a few hours; (2) horizontal spontaneous nystagmus often with a rotational component toward the unaffected ear during a vertigo attack without evidence of a central vestibular lesion; (3) caloric test showing hyporesponsiveness or lack of responsiveness of the horizontal canal of the affected ear (canal paresis (CP) ≥ 50% on the affected side); and (4) no cochlear signs or other neurological signs. The percentage of CP diagnosed as VN varies according to the report. The use of video head impulse tests rather than caloric tests to evaluate CP related to VN is becoming more common [14]. According to reports, the video head impulse test is abnormal in caloric tests with CP ≥ 50% (50% [15], and 52.5% [16]). In this study, we diagnosed patients with VN whose CP on the affected side was ≥ 50% to ensure easy comparison with other reports.

Data collection

Baseline parameters included age, sex, height (cm), weight (kg), body mass index (BMI, kg/m^2^), systolic blood pressure (SBP, mmHg), diastolic blood pressure (DBP, mmHg), heart rate (bpm), CAVI, diabetes mellitus rate (%), hypertension rate (%), dyslipidemia rate (%), and rate of history of diabetes mellitus, hypertension, or dyslipidemia (%). Diabetes mellitus was defined as fasting plasma glucose ≥ 126 mg/dL and/or 75-g oral glucose tolerance test with 2 h plasma glucose ≥ 200 mg/dL and/or random plasma glucose ≥ 200 mg/dL. Hypertension was defined as SBP ≥ 140 mmHg and/or DBP ≥ 90 mmHg. Dyslipidemia was defined as a total cholesterol level ≥ 220 mg/dL, high-density lipoprotein cholesterol level < 40 mg/dL, and/or triglyceride level ≥ 150 mg/dL. Moreover, patients treated with antidiabetic, antihypertensive, or lipid-lowering agents were diagnosed and included.

Measurement and evaluation of the vestibular function

A bithermal caloric test, which evaluates the lateral semicircular canal function, was performed using 46°C warm and 26°C cold air for 60 s at the initial examination, once every two weeks until two months after onset, once one to two months until six months after onset, and once three to four months until their CP stabilized or their complete CP recovery. CP was calculated using the following formula using the maximum slow-phase eye velocity of induced nystagmus based on the Jangkees formula [17]:

Percent CP = 100 |(RC+RW)-(LC+LW)|/(RC+RW+LC+LW)

RW: the maximum slow-phase eye velocity of induced nystagmus when the right ear is stimulated with warm air; RC: the right ear with cold air; LW: the left ear with warm air; LC: the left ear with cold air

Tests were performed using an electronystagmography (Nystagmograph NY-50, RION Co. Ltd., Tokyo, Japan) in the supine position (head up 30°) in a completely dark room. The percentage of CP diagnosed as VN varies by report and is approximately ≥25% (22% [18], 25% [19], 25-27%, and 30% [16]). In this study, we employed quartiles and set to evaluate the severity of CP as no CP (intact, 0% ≤ CP < 25%), mild CP (25% ≤ CP < 50%), moderate CP (50% ≤ CP < 75%), and severe CP (75% ≤ CP ≤ 100%). Patients with moderate CP were diagnosed with moderate VN and those with severe CP with severe VN (Table 1).

**Table 1:** Criteria for the grading of percent CP and the severity of vestibular neuritis CP: canal paresis

	Grading of percent CP	Severity of vestibular neuritis
no CP (intact)	0% ≤ CP < 25%	
mild CP	25% ≤ CP < 50%	
moderate CP	50% ≤ CP < 75%	moderate vestibular neuritis
severe CP	75% ≤ CP ≤ 100%	severe vestibular neuritis

Furthermore, the improvement criteria for VN vary among reports. In this study, the level of horizontal semicircular canal function recovery was defined as recovery, improvement, and no change including deterioration (Table 2).

**Table 2:** Level of recovery in percent CP CP: canal paresis

Level of recovery	Change in CP
Complete recovery	severe or moderate to no CP
Improvement	severe to moderate or mild, or moderate to mild CP
No change (including deterioration)	severe to severe, moderate to moderate, or moderate to severe CP

Vestibular-evoked myogenic potentials (VEMP) [20], which evaluate saccular function, were performed in response to short tone bursts (500 Hz, rise/fall time 1 ms, plateau time 2 ms, 95 dB nHL; burst-VEMP) using Neuropack Σ (Nihon Kohden, Tokyo, Japan). Surface electromyographic activity was recorded from symmetrical sites over the upper half of each sternocleidomastoid muscle (SCM) with reference electrodes on the lateral end of the upper sternum. The ground electrode was placed on the nasion. During the recording of the burst-VEMP, patients were instructed to raise their heads to activate the SCM, and electromyography of the bilateral SCM was monitored. The responses of the VEMP were regarded as abnormal when the responses on the affected side were absent or decreased compared with those on the unaffected side. For this comparison, the percentage VEMP asymmetry [20] was calculated using the following formula:

Percent VEMP asymmetry = 100 | (Au - Aa) | / (Au + Aa)

Aa: the amplitude of p13-n23 on the affected side; Au: the amplitude on the unaffected side The upper limit of percent VEMP asymmetry was set as 34.1 according to a previous report [20].

Measurement and evaluation of CAVI

CAVI was measured with a VaSera CAVI instrument (Fukuda Denshi Inc., Tokyo, Japan) by the methods described elsewhere [10]. In brief, cuffs were applied to the bilateral upper arms and ankles with the subject lying supine and the head held in the midline position. After resting for 10 min, to detect brachial and ankle pulse waves with cuffs, a low cuff pressure of 30-50 mmHg was used to ensure the minimal effect of cuff pressure on hemodynamics. Blood pressure was measured using cuffs on the upper arm.

The CAVI was calculated based on the stiffness parameter β theory [21] using the following formula: CAVI = a{(2ρ/∆P) × ln(Ps/ Pd)PWV2}+b

Ps: systolic blood pressure; Pd: diastolic blood pressure; PWV: pulse wave velocity; ∆P: Ps-Pd; ρ: blood density; a and b: constants

PWV was obtained by dividing the vascular length by the time taken for the pulse wave to propagate from the aortic valve to the ankle and was measured using cuffs at the upper arms and ankles. The mean coefficient of variation of CAVI is 3.85 [10], and CAVI has good reproducibility [22] for clinical use because the accepted limit for clinical laboratory testing is 5%.

CAVI = 8.0 is reported to be an optimal cutoff point for carotid arteriosclerosis prediction [23]. The group with a CAVI > 10 is reported to have a high incidence of heart diseases and cerebrovascular accidents in three years [24]. Meanwhile, based on data from healthy subjects (15,966 females and 16,661 males), the standard value for CAVI on the measuring instrument was set as ≤ 8.9 on the measuring instrument. Therefore, we defined low CAVI as 8.9 or lower and high CAVI as > 8.9.

Statistical analysis

Statistical Package for the Social Sciences (IBM SPSS Statistics for Windows, IBM Corp., Version 22.0, Armonk, NY) was used for statistical analysis. The sample size was calculated based on the percentages of the level of hearing recovery (no change versus others) between high and low CAVI patients with a one-sided alpha level of 0.05 and a statistical power of 80%. Correlations between age (years), height (cm), weight (kg), body mass index (BMI), blood pressure (mmHg), heart rate (bpm), initial and final severity of CP (%), presence or absence of VEMP asymmetry, time to percent CP stabilized or recovery (days), level of percent CP recovery, and CAVI (normal: < 8.9; abnormal: ≥ 8.9) were examined using simple linear regression analysis and unpaired Welch’s t-test for continuous variables, and chi-square test for categorical variables. The included patients were fitted to the Cox proportional hazard model with recovery as the outcome. For the multivariate analysis, significant variables in the univariate analysis were used as covariates.

Results are presented as the mean ± standard deviation, and statistical significance was set at p < 0.05.

## Results

**Figure 1:**
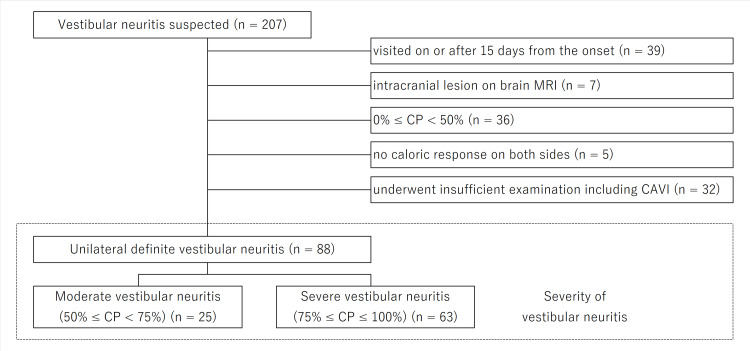
Patients’ description CP: canal paresis, CAVI: cardio-ankle vascular index

The sample size was calculated based on the percentage of recovered patients with CP between patients with high and low CAVI. Finally, 88 of the 207 patients who were suspected of VN (31 high CAVI and 57 low CAVI patients; 59.3 ± 15.3 years; 41 females and 47 males) and enrolled with a one-sided alpha level of 0.05 and an actual statistical power of 80.9%. Exclusion criteria included the following: patients visited on or after 15 days from the onset or had been treated with steroids at a previous clinic (n = 39), patients were diagnosed with an intracranial lesion on brain magnetic resonance imaging (n = 7), had CP < 50% (n = 36), patients had no caloric response on either side (n = 5), and underwent insufficient examination (n = 32) (Figure 1).

Patients with no caloric response on either side were excluded from this study because they did not fit the diagnostic criteria of a previous report [5]. Furthermore, they might have bilateral vestibular dysfunction or vestibular dysfunction on the opposite side.

The effect of steroids on improving vestibular function remains controversial [25], although methylprednisolone has been reported to significantly improve the recovery of peripheral vestibular function in patients with VN [7]. In this study, all patients were given a short course of corticosteroids. The dose was tapered from prednisolone 1 mg/kg per day, with a maximum dose of 60 mg/day to 10 mg/day over 10 days.

Comparison of patients with low CAVI and those with high CAVI

**Figure 2:**
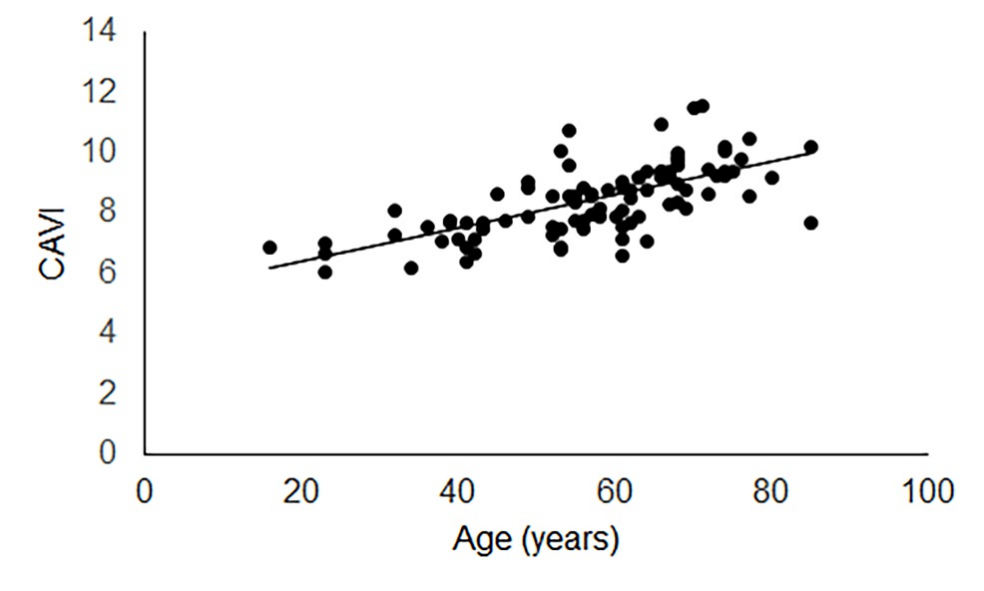
Regression of age to CAVI on the unaffected side Age showed a positive regression to CAVI on the unaffected side (y = 0.055x + 5.32, R² = 0.44, p < 0.001). CAVI: cardio-ankle vascular stiffness index

**Figure 3:**
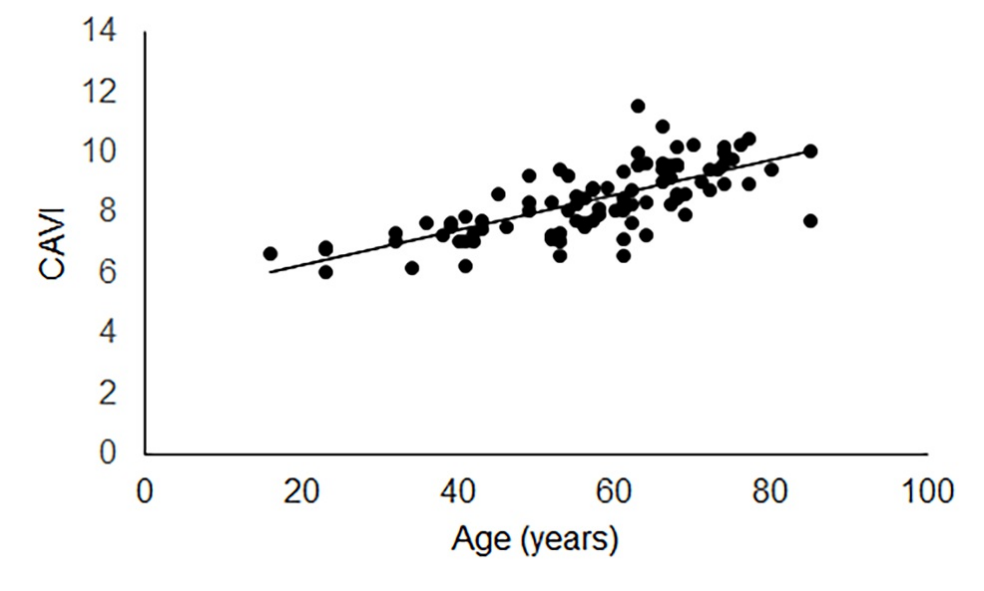
Regression of age to CAVI on the affected side Age showed a positive regression to CAVI on the affected side (y = 0.058x + 5.16, R² = 0.51, p < 0.001). CAVI: cardio-ankle vascular stiffness index

In 88 patients enrolled, the initial caloric test was undergone 4.2 ± 4.8 days after VN onset. CAVI (57 low CAVI cases and 31 high CAVI cases) was undergone 3.4 ± 4.6 days after the initial caloric test. Age showed a positive regression to CAVI on both the unaffected (Figure 2) and affected (Figure 3) sides.

The age and SBP of the high CAVI group were higher than those of the low CAVI group at the initial examination. The initial percentage of CP was almost the same in the high and low CAVI groups. The final percentage of CP tended to be lower in the high CVI group than in the low CAVI group. The percentage of the presence or absence of VEMP asymmetry and medical history was almost the same in the low and high CAVI groups. Time to percent CP stabilized or recovery tended to be longer in the low CAVI group than in the high CAVI group (Table 3).

**Table 3:** Characteristics and test results of the low CAVI and high CAVI groups Values are shown as mean ± SD (standard deviation). n: number of patients; BMI: body mass index; CAVI: cardio-ankle vascular index; CP: canal paresis; VEMP: vestibular evoked myogenic potentials †Unpaired t-test, ††Welch's t-test, ‡chi-square test, ‡‡Yates's chi-square test *: p <0.05, **: p < 0.01

	Low CAVI group (n = 57)	High CAVI group (n = 31)	p
Age (years)	51.7 ± 13.7	67.9 ± 7.9	< 0.001^†**^
Gender			
female	27 (47.4%)	14 (45.2%)	0.25^‡^
male	30 (52.6%)	17 (54.8%)	
height (cm)	162.6 ± 9.4	161.2 ± 8.6	0.47^†^
weight (kg)	62.8 ± 11.8	60.7 ± 9.6	0.41^†^
BMI (kg/m^2^)	23.6 ± 3.1	23.2 ± 2.1	0.52^††^
Blood pressure (mmHg)			
systolic	132.9 ± 17.9	141.9 ± 22.9	0.045^†*^
diastolic	82.1 ± 11.7	82.6 ± 11.5	0.86^†^
Heart rate (bpm)	64.7 ± 10.7	66.9 ± 9.4	0.33^†^
CAVI	7.79 ± 0.73	9.77 ± 0.69	< 0.001^†**^
Present medical histories			
Diabetes mellitus	3 (5.3%)	6 (19.4%)	0.086^‡‡^
Hypertension	15 (26.3%)	10 (32.3%)	0.55^‡^
Dyslipidemia	8 (14.0%)	5 (16.1%)	1.00^‡‡^
Any of above histories	20 (35.1%)	14 (45.2%)	0.35^‡^
Initial percent CP (%)	85.14 ± 344.17	87.19 ± 273.11	0.54^†^
Final percent CP (%)	43.56 ± 32.17	34.50 ± 30.81	0.21^†^
Present VEMP asymmetry	8 (14.0%)	5 (16.1%)	1.00^‡‡^
Time until percent CP stabilized or recovery (days)	201.3 ± 148.8	174.8 ± 116.0	0.41^†^

**Figure 4:**
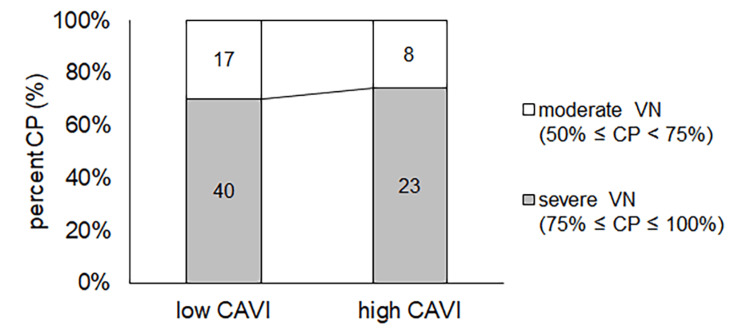
The number of moderate and severe VN patients in the low CAVI and high CAVI groups The number of moderate and severe VN was 17 and 40 patients in the low CAVI group and 8 and 23 patients in the high CAVI group, respectively. The percentage of moderate and severe VN in the high CAVI group was almost the same as that in the low CAVI group (p = 0.69). CAVI: cardio-ankle vascular stiffness index; VN: vestibular neuritis, CP: canal paresis

The percentages of moderate and severe VN in the high CAVI group were almost the same as those in the low CAVI group (p = 0.69) (Figure 4).

**Figure 5:**
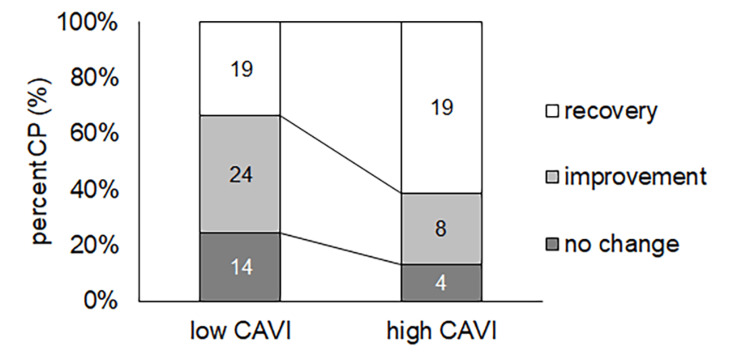
The number of recovered, improved, and no changed patients in the low CAVI and high CAVI groups The number of recovered, improved, and no changed patients were 19, 24, and 14 patients in the low CAVI group and 19, 8, and 4 cases in the high CAVI group, respectively. The percentage of each outcome was different (p = 0.04), and the percentage of recovered patients in the high CAVI group was higher than that in the low CAVI group (p = 0.011). CAVI: cardio-ankle vascular stiffness index

The percentages of moderate and severe VN in the high CAVI group were almost the same as those in the low CAVI group (p = 0.69) (Figure 4). In contrast, the percentage of each outcome was different (p = 0.040), and the percentage of recovered patients in the high CAVI group was higher than that in the low CAVI group (p = 0.011) (Figure 5).

Comparison of the patients with recovered and others in percent CP

In univariate analyses, the age of the patients who recovered in percent CP was not different from those with improved and unchanged in percent CP. The percentage of patients with severe VN among the recovered patients was higher than that among the others. The percentage of patients with high CAVI among the recovered patients was higher than that among the others. The percentage of patients with present VEMP asymmetry and having past medical histories in patients with low CAVI was not different from those with high CAVI. The time to percentage CP stabilization or recovery was longer in the low CAVI group than in the high CAVI group (Table 4).

**Table 4:** Univariate analyses of factors between a complete recovery or marked improvement in hearing patients and slight improvement or no change in patients Values are shown as mean ± SD (standard deviation). n: number of patients; CP: canal paresis; CAVI: cardio-ankle vascular index; VEMP: vestibular evoked myogenic potentials †Unpaired t-test, ††Welch's t-test, ‡chi-square test, ‡‡Yates's chi-square test *: p <0.05, **: p < 0.01

	Recovery in CP (n = 38)	Improvement or no change in CP (n = 50)	p
Age (years)	58.1 ± 16.0	57.3 ± 13.0	0.9^†^
Gender			
female	18 (47.4%)	23 (46.0%)	0.25^‡^
male	20 (52.6%)	27 (54.0%)	
Severity of VN			
moderate VN	17 (44.7%)	8 (16.0%)	0.0031^‡**^
severe VN	21 (55.3%)	42 (84.0%)	
CAVI			
low	19 (50.0%)	38 (76.0%)	0.011^‡*^
high	19 (50.0%)	12 (24.0%)	
VEMP asymmetry			
absent	35 (92.1%)	40 (80.0%)	0.11^‡^
present	3 (7.9%)	10 (20.0%)	
Present medical histories			
Diabetes mellitus	3 (7.9%)	6 (12.0%)	0.78^‡‡^
Hypertension	10 (26.3%)	15 (30.0%)	0.7^‡^
Dyslipidemia	7 (18.4%)	6 (12.0%)	0.4^‡^
Any of above histories	16 (42.1%)	18 (36.0%)	0.56^‡^
Time to percent CP stabilized or recovery (days)	145.3 ± 103.0	227.9 ± 151.3	0.0041^††*^

For multivariate analyses, we set three covariates because of the lower number of patients in the two groups (n = 38). The severity of VN and CAVI were set as covariates because they were significant in univariate analysis. Age was also set because CAVI and age could be confounders of each other, as they were positively correlated. In the Cox proportional hazard model contributing to the recovery in percent CP as the outcome, high CAVI, less severe VN, and lower age were significant covariates (Table 5).

**Table 5:** Results of the multivariate analysis fitted to the Cox proportional hazards model contribute to recovery in percent CP HR: hazard ratio; CI: confidence interval; ref: reference; CP: canal paresis; VN: vestibular neuritis; CAVI: cardio-ankle vascular index *: p <0.05, **: p < 0.01

	HR	95% CI	p
Age			
increases 1 year	0.97	0.94-0.99	0.013^*^
Severity of VN			
moderate VN (ref)			
severe VN	0.28	0.14-0.55	< 0.001^**^
CAVI			
low (ref)			
high	3.95	1.67-9.39	0.0018^**^

## Discussion

In this study, high CAVI, less severe CP, and younger age were identified as variables for semicircular canal function improvement in VN. Moreover, despite older age and the same severity of VN, semicircular canal function improved better in patients with high CAVI than in those with low CAVI. In other words, patients with high CAVI, or stiffer and less elastic arteries, have a better prognosis than their counterparts.

Diseases in patients with stiff and low elastic arteries are expected to be difficult to recover from, for example, more advanced or more severe lesions were observed in cardiovascular [13], cerebrovascular [12], and macular disorders [26] with high CAVI. Contrarily, in our study, VN exhibited a better prognosis in patients with high CAVI than those with low CAVI. To explain the discrepancy, we hypothesized that the different percentages of etiologies are included in the high CAVI and low CAVI patients, i.e., the percentage of VN due to vascular disorders in high CAVI patients is higher than that due to viral infection because similar prognoses are expected for VN with the same etiology and severity of VN. Furthermore, the time to percent CP stabilized or recovery was longer in low CAVI patients than in the high CAVI patients, suggesting VN with high CAVI and better prognosis was “more” transient, i.e., vascular disorders.

Another hypothesis is that both groups had VN due to viral infection; however, patients with a high CAVI had more severe vascular changes due to viral inflammation. An increased percentage of CD40-positive monocytes and macrophages, which can lead to thrombotic and inflammatory changes in the vessels, was reported in patients with VN compared to healthy individuals [27]. These pro-inflammatory activations can reduce the microvascular perfusion of the inner ear organs caused by an increase in thrombotic events [28]. However, if all VN is caused by a viral infection, then the claim that semicircular canal function has a better prognosis in patients with more severe vascular changes is contradictory. In any case, the percentage of VN due to vascular disorders is higher in patients with high CAVI than in those with low CAVI; therefore, better improvement in vestibular function was achieved in high CAVI patients.

As for the correlation between the severity and the prognosis of VN, some reports have shown that the prognoses of the patients with no response in the caloric test at the first examination were not always bad [29]. However, others showed the more severe the VN is, the worse the prognosis of the semicircular function is [18], which was consistent with the findings of this study showing that the VN severity was significant both on univariate and multivariate analyses. Contrarily, in this study, age was significant only in multivariate analysis. The reason this discrepancy was observed in age can be explained as follows: CAVI and age were confounders of each other, as they had positive regression. However, the prognosis of VN worsens with increasing age, and conversely, it improves with increasing CAVI. Multivariate analysis resolved confounding factors, and age was a significant covariate, in addition to CAVI. Some reports have shown a correlation between the prognosis of VN and age [18], which is consistent with the findings of this study; while few other studies have shown no such correlation [29]. The result of VEMP asymmetry was consistent with a previous report showing that present or absent VEMP asymmetry was not associated with VN prognosis [30]. Moreover, past medical histories were not associated with VN prognosis. We can conclude that VEMP asymmetry and past medical histories were not as prognostic as CAVI, VN severity, and age.

This study has some limitations. First, we cannot conclude from our results that VN in high CAVI patients is due to vascular disorders; therefore, semicircular canal function improved better, while VN in low CAVI patients was due to viral infection, and semicircular canal function improved worse. This is because CAVI is an index of vascular stiffness and elasticity and cannot directly determine the etiology. Second, all enrolled patients did receive a short steroid treatment; therefore, it might be inappropriate to draw inferences about natural disease progression.

## Conclusions

VN in patients with high CAVI had a better prognosis in semicircular canal function than in patients with low CAVI, despite higher age and the same severity of VN. On multivariate analysis, high CAVI, less severe CP, and younger age were factors for better improvement in semicircular canal function. We believe that CAVI can be an additional indicator for estimating the prognosis and etiology of VN, although further studies are required to confirm this.
